# Intense Acute Swimming Induces Delayed-Onset Muscle Soreness Dependent on Spinal Cord Neuroinflammation

**DOI:** 10.3389/fphar.2021.734091

**Published:** 2022-01-07

**Authors:** Sergio M. Borghi, Sylvia K. D. Bussulo, Felipe A. Pinho-Ribeiro, Victor Fattori, Thacyana T. Carvalho, Fernanda S. Rasquel-Oliveira, Tiago H. Zaninelli, Camila R. Ferraz, Antônio M. B. Casella, Fernando Q. Cunha, Thiago M. Cunha, Rubia Casagrande, Waldiceu A. Verri

**Affiliations:** ^1^ Departamento de Ciências Patológicas, Centro de Ciências Biológicas, Universidade Estadual de Londrina, Londrina, Brazil; ^2^ Centro de Pesquisa Em Ciências da Saúde, Universidade Norte do Paraná, Londrina, Brazil; ^3^ Departamento de Clínica Médica, Centro de Ciências da Saúde, Universidade Estadual de Londrina, Londrina, Brazil; ^4^ Departamento de Farmacologia, Faculdade de Medicina de Ribeirão Preto, Universidade de São Paulo, São Paulo, Brazil; ^5^ Departamento de Ciências Farmacêuticas, Centro de Ciências de Saúde, Hospital Universitário, Universidade Estadual de Londrina, Londrina, Brazil

**Keywords:** acute exercise, delayed-onset muscle soreness, spinal cord, glial cells, neuroinflammation

## Abstract

Unaccustomed exercise involving eccentric contractions, high intensity, or long duration are recognized to induce delayed-onset muscle soreness (DOMS). Myocyte damage and inflammation in affected peripheral tissues contribute to sensitize muscle nociceptors leading to muscle pain. However, despite the essential role of the spinal cord in the regulation of pain, spinal cord neuroinflammatory mechanisms in intense swimming-induced DOMS remain to be investigated. We hypothesized that spinal cord neuroinflammation contributes to DOMS. C57BL/6 mice swam for 2 h to induce DOMS, and nociceptive spinal cord mechanisms were evaluated. DOMS triggered the activation of astrocytes and microglia in the spinal cord 24 h after exercise compared to the sham group. DOMS and DOMS-induced spinal cord nuclear factor κB (NFκB) activation were reduced by intrathecal treatments with glial inhibitors (fluorocitrate, α-aminoadipate, and minocycline) and NFκB inhibitor [pyrrolidine dithiocarbamate (PDTC)]. Moreover, DOMS was also reduced by intrathecal treatments targeting C-X_3_-C motif chemokine ligand 1 (CX_3_CL1), tumor necrosis factor (TNF)-α, and interleukin (IL)-1β or with recombinant IL-10. In agreement, DOMS induced the mRNA and protein expressions of CX_3_CR1, TNF-α, IL-1β, IL-10, c-Fos, and oxidative stress in the spinal cord. All these immune and cellular alterations triggered by DOMS were amenable by intrathecal treatments with glial and NFκB inhibitors. These results support a role for spinal cord glial cells, *via* NFκB, cytokines/chemokines, and oxidative stress, in DOMS. Thus, unveiling neuroinflammatory mechanisms by which unaccustomed exercise induces central sensitization and consequently DOMS.

## Introduction

The perception of muscle pain occurs because of sensitization and/or activation of group III (Aδ-fiber) and group IV (C-fiber) polymodal muscle afferents (nociceptors) in peripheral sites after overload and injuries in skeletal myocytes. Nociceptor neuron sensitization reduces its mechanical threshold to weak non-nociceptive stimuli or enhances neuronal activation to nociceptive stimuli leading to allodynia or hyperalgesia, respectively (Connolly et al., 2003; Graven-Nielsen and Arendt-Nielsen, 2003; Kubo et al., 2012; Matsubara et al., 2019). Signs of exercise-induced muscle pain become apparent between 24 and 48 h after the practice [delayed-onset muscle soreness (DOMS)] in response to unaccustomed exercise, especially those related to muscle fatigue, or of high intensity and long duration (MacIntyre et al., 1995; Taylor et al., 2000; Cheung et al., 2003; Graven-Nielsen and Arendt-Nielsen, 2003; Dannecker and Koltyn, 2014).

Mechanical and/or chemical stimuli detected by peripheral terminals of afferent muscle fibers are then transmitted to spinal cord sites and, subsequently, to higher nervous centers, such as brainstem, thalamus, and somatosensory cortex, where they will be processed (Basbaum et al., 2009). The cell body of primary sensory neurons is localized in the dorsal root ganglia (DRG) and has both peripheral and central axonal branches that innervate skeletal muscle at one extremity and reach the spinal cord at the other (Basbaum et al., 2009). The central axonal branch of muscle nociceptors projects to the spinal cord through the dorsal horn, performing synapses with neurons in laminas I, II and lamina V (Hoheisel et al., 1994; Panneton et al., 2005). In these foci, neuronal–glial interactions account for the central sensitization in several muscle pain conditions. In line with this, it was demonstrated that the depolarization of peripheral nociceptors induces the secretion of the chemokine C-X_3_-C motif chemokine ligand 1 (CX_3_CL1; also known as fractalkine) by DRG neurons, which binds to C-X_3_-C motif receptor 1 (CX_3_CR1) in spinal microglia at these sites, stimulating these cells to produce cytokines [such as interleukin-1β (IL-1β)], prostaglandin E2 (PGE_2_), and brain-derived neurotrophic factor (BDNF), promoting increasing excitability and enhanced pain in response to noxious stimuli in neuropathic and inflammatory pain conditions (Milligan et al., 2004; Basbaum et al., 2009; Gao and Ji, 2010; Souza et al., 2013). As a major pro-inflammatory transcription factor, nuclear factor κB (NFκB) also plays a critical role during central sensitization after peripheral damage (Lee et al., 2004; Souza et al., 2015; Liu et al., 2018). All these data raise the assumption that increased nociceptive activity projected from overloaded skeletal muscle induces central effects in muscle pain-like conditions, including those induced by intense exercise.

Studies focusing on peripheral events in DOMS protocols are largely found in the literature (Cheung et al., 2003; Murase et al., 2010; Murase et al., 2013; Borghi et al., 2014a; Borghi et al., 2014b); however, the mechanisms related to spinal cord sensitization in this condition still remain poorly investigated. Evidence applying exercise models of eccentric (8-week training of downhill running) (Pereira et al., 2015) and aerobic (treadmill running; 40 min) (Dos Santos et al., 2020) characteristics to induce DOMS observed spinal cord dorsal horn glial reactivity from 24 to 36 h in post-exercise recovery period. Data regarding the mechanisms in DOMS may vary a lot depending on the exercise protocol applied, thus supporting the need of evaluating varied exercise protocols for a better general interpretation of central responses to acute intense exercise. Therefore, in the present study, we addressed the role of spinal glial cells (astrocytes and microglia) and the mechanisms by which these cells modulate exercise-induced neuroinflammation and pain using a model of intense acute swimming-induced muscle mechanical hyperalgesia in mice as we previously described (Borghi et al., 2014a; Borghi et al., 2014b; Borghi et al., 2015; Borghi et al., 2016). Models using classical eccentric exercise protocols are common to study DOMS pathophysiological mechanisms; however, the investigation of models characterized by exercise protocols with high intensity and long duration is less frequent. We developed an intense acute swimming protocol to fill this gap (Borghi et al., 2014b). We hypothesized that spinal cord glial cells, such as astrocytes and microglia, have a role in DOMS-induced neuroinflammation, whereas they trigger inflammatory mechanisms in response to peripheral stimuli. Our hypothesis differs from previous data in the sense that we searched for a causal relationship between gliosis and pain (Pereira et al., 2015) and questioned whether the activation of peripheral nociceptor neurons could also be a contributing mechanism to spinal cord gliosis and pain and not solely an inflammatory molecule produced during exercise such as heat shock protein (Hsp)70 (Dos Santos et al., 2020).

## Materials and Methods

### Animals

The experiments were performed on male pathogen-free C57BL/6 and heterozygous CX_3_CR1-eGFP^+/-^ C57BL/6 mice [replacement of CX_3_CR1 gene expression by green fluorescent protein (GFP) marker], 12 weeks, weighing between 20 and 25 g from the State University of Londrina (UEL), Paraná, Brazil, and Ribeirao Preto Medical School, University of Sao Paulo (USP), São Paulo, Brazil, respectively. Only male mice were used because of the well-known sex dimorphism in pain regulation in this species (Sorge et al., 2015; Chen et al., 2018). Mice were placed in standard clear plastic cages of ventilated rack (Alesco Indústria e Comércio LTDA, Monte Mor, São Paulo, Brazil) and fed *ad libitum*, light/dark cycle of 12/12 h, and temperature-controlled room. Mice were maintained in the vivarium of the Department of Pathology of UEL for at least 2 days before the experiments. Mice were used only once and were acclimatized to the testing room by at least 1 h before the behavioral experiments, which were conducted exclusively during the light cycle. At the end of the experiments, mice were anesthetized with isoflurane 5% only once by inhalation overdose and terminally killed by cervical dislocation followed by decapitation. Animals’ care and handling procedures were in accordance with the International Association for Study of Pain (IASP) guidelines and with the approval of the Institutional Ethics Committee for Animal Research of UEL (CEUA-UEL), process number 4010.2015.27. All efforts were made to minimize the number of animals used and their suffering. No unexpected deaths occurred during the study. Animals were identified and subsequently randomized using a true random number service.

### Chemicals

The pharmacological compounds used in this study are presented in Supplementary Table S1 that also shows the intended use and range of doses administered.

### Methods for Intrathecal Injections

**FIGURE 1:**
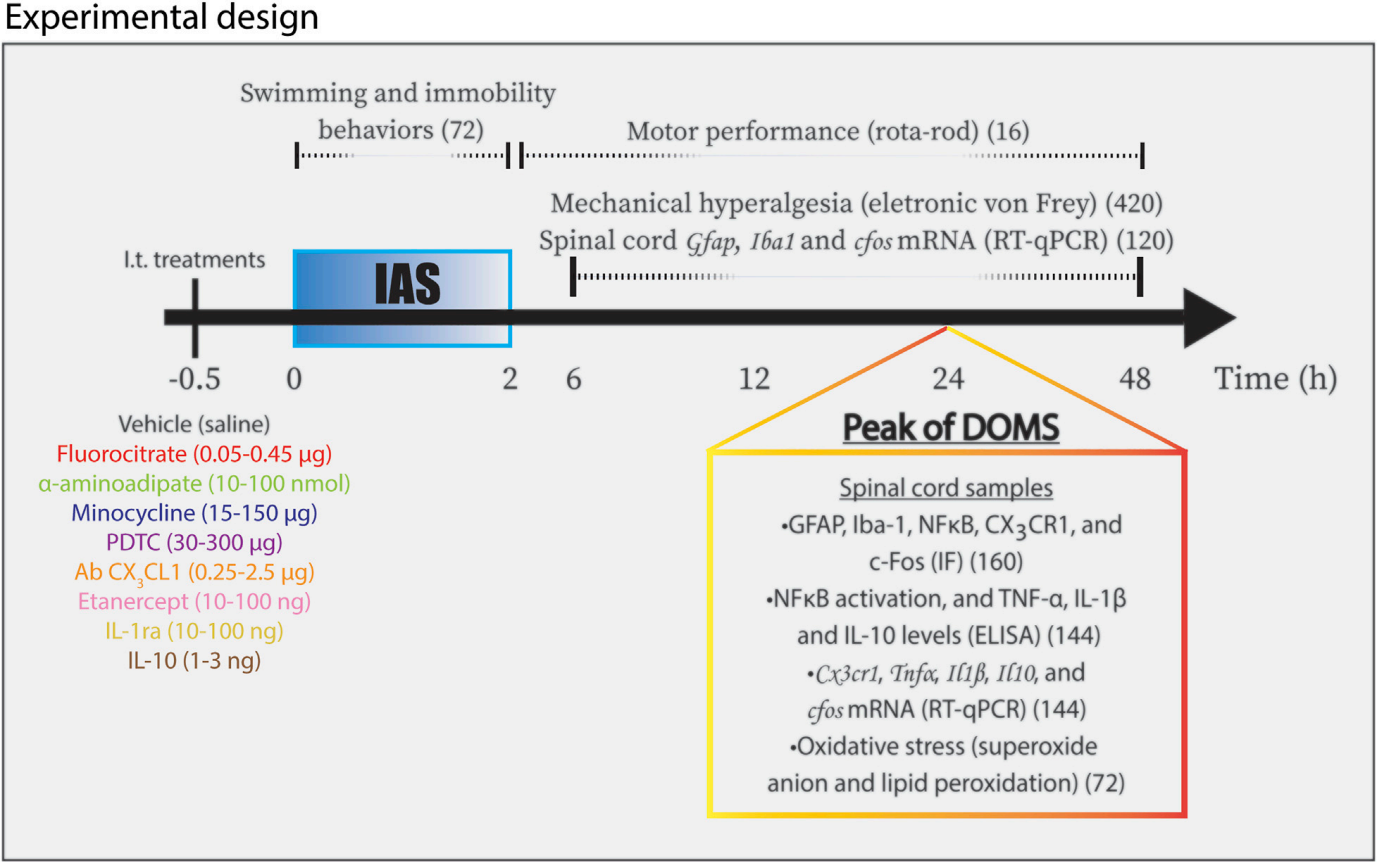
Schematic representation of experimental design used in the study. Mice were randomly divided in different experimental groups receiving i.t. treatments according to targets 30 min (−0.5 h in diagram) before IAS. Mice experienced IAS session for two consecutive hours (0–2 h in diagram). Swimming behavior and immobility behavior were evaluated during the session. Motor performance analysis was conducted between 30 min and 48 h after the session. The measurements of mechanical hyperalgesia and the determination of spinal cord *Gfap*, *Iba1*, and *cfos* mRNA expression were carried out from 6 to 48 h after the IAS session. In the peak of DOMS (24 h in diagram), spinal cord samples were collected for the following approaches: GFAP, Iba-1, pNFκB, CX_3_CR1, and c-Fos for IF analysis; ELISA tests for NFκB activation and TNF-α, IL-1β, and IL-10 protein levels; *Cx3cr1*, *Tnfα*, *Il1β*, and *Il10* mRNA expression determination by RT-qPCR; and oxidative stress (superoxide anion production and lipid peroxidation levels) evaluation. IAS, intense acute swimming; IF, immunofluorescence. The total number of mice per analysis is shown within parentheses.

The administration of drugs by intrathecal (i.t.) route was performed in unconscious animals (targeting lumbar segment, L_4_–L_6_ zone) under anesthesia with isoflurane 3% inhalation (Abbott Park, IL, USA), randomly divided in treatment groups described in Figure 1. Fluorocitrate (0.05–0.45 µg), α-aminoadipate (10–100 nmol), minocycline (15–150 µg), and pyrrolidine dithiocarbamate (PDTC; 30–300 µg) were diluted in dimethyl sulfoxide (DMSO; D8418; Sigma-Aldrich, St. Louis, MO, USA), Tween 20% (P1379; Sigma-Aldrich, St. Louis, MO, USA), and saline. Antibody anti-CX_3_CL1 (0.25–2.5 g), etanercept (10–100 ng), IL-1ra (10–100 ng), and mouse recombinant (mr) IL-10 (1–3 ng) were diluted only in saline. The dilutions were made immediately before i.t. administration of the drugs. After performing the dose–response experiment (Supplementary Table S1), the doses of 0.15 µg of fluorocitrate, 100 nmol of α-aminoadipate, 150 µg of minocycline, and 300 µg of PDTC were chosen for the next sets of experiments. I.t. injections were performed to achieve a local effect on spinal sites. Mice received i.t. treatments always 30 min before intense acute swimming (IAS). I.t. treatments were performed only once to avoid excessively injuring local tissues, which would cause inflammation and enhanced nociceptive responses (Almeida et al., 2000).

### General Experimental Procedures

Mice were divided into 22 experimental groups during the study, as follows: sham; IAS; IAS + vehicle (saline); IAS + fluorocitrate 0.05, 0.15, and 0.45 µg; IAS + α-aminoadipate 10, 30, and 100 nmol; IAS + minocycline 15, 50, and 100 μg; IAS + PDTC 30 and 300 μg; IAS + Ab CX_3_CL1 0.25 and 2.5 μg; IAS + etanercept 10 and 100 ng; IAS +10 and 100 ng; and IAS + mrIL-10 1 and 3 ng. We used 6 mice per group in each experiment per analysis apart from immunofluorescence, motor performance and time spent in swimming behavior and immobility time in which 4 mice were used per group per experiment. Every experiment was performed twice. Prior studies with the IAS model and similar experimental parameters were used to define the *n* for each group (Borghi et al., 2019; Borghi et al., 2021). Sham animals (exposed to water during 30 s only) were used as control group of IAS. Figure 1 summarizes the experimental design and total number of animals used in each experiment. Total number of animals used in the present study was 1,148. Mice were treated by i.t. route always 30 min before the IAS session. The following time points were used for the indicated evaluation parameters: time–response of mRNA expression of glial fibrillary acidic protein (GFAP) and ionized calcium-binding adaptor molecule 1 (Iba-1) (biomarkers for astrocytic and microglial activity, respectively) in spinal cord samples (6–48 h after IAS); spinal cord immunofluorescence assay for GFAP and Iba-1 at the peak of its mRNA expression (24 h after IAS); muscle mechanical hyperalgesia (6–48 h after IAS); spinal cord CX_3_CR1-eGFP fluorescence and spinal cord NFκB activation (ELISA and immunofluorescence, 24 h after IAS); spinal cord mRNA expression of *Cx3cr1*, *Tnfα*, *Il1β*, and *Il-10* (RT-qPCR, 24 h after IAS); spinal cord protein levels of tumor necrosis factor (TNF)-α, IL-1β, and IL-10 (ELISA, 24 h after IAS); spinal cord oxidative stress (superoxide anion production and lipid peroxidation levels) (colorimetric tests, 24 h after IAS); and spinal cord *cfos* mRNA expression (RT-qPCR, 6–48 h after IAS) and protein staining (immunofluorescence, 24 h after IAS) (Borghi et al., 2014b). All these analyses were performed with samples of the spinal cord lumbar segment (L_4_–L_6_). Motor function of animals was analyzed using the rotarod performance test, which did not identify impairment of motor capacity (0.5–48 h after IAS; Supplementary Figure S1A). Time spent in swimming behavior and immobility time behavior during unaccustomed IAS session were evaluated (in minutes; Supplementary Figures S1B, C, respectively) in vehicle, α-aminoadipate, minocycline, fluorocitrate, PDTC, antibody anti-CX_3_CL1, etanercept, IL-1ra- and IL-10-treated groups to determine whether the doses used in the study interfered in the swimming behavior, and consequently, in the nociceptive, biochemical, and molecular analyses. None of the treatments altered the time spent swimming or the immobility time of the mice (Supplementary Figures S1B, C), thus confirming that behavior and immune-biochemical changes observed were due to the involvement of such mechanisms in IAS and not by altered time of swimming. The ARRIVE guideline checklist can be found in supplementary information (Kilkenny et al., 2010).

### Unaccustomed Intense Acute Swimming Protocol

Mice were placed in a glass box (45 cm × 28 cm × 25 cm, divided into six compartments) with approximately 20 L of temperature-controlled water at 31°C ± 1°C. Each mouse was placed alone in individual compartments at the same time as for all mice. Sham animals were allowed to swim for just 30 s and were immediately removed from the water after this period. Mice in the swimming group were exposed to water for one acute session with a duration of 2 h. The muscle mechanical hyperalgesia was evaluated 6–48 h after the IAS session. The present study used a model of swimming exercise, avoiding stress or hypoalgesia, and focusing on exercise-induced hyperalgesia as previously demonstrated and standardized (Kuphal et al., 2007; Borghi et al., 2014b).

### Motor Performance Assessment

To evaluate whether IAS session affects motor function of mice, motor performance was evaluated using the rotarod performance test. The test is used to quantitate the effects of varied conditions and procedures upon motor planning with very high reliability (Rustay et al., 2003). The apparatus consists of a bar with a diameter of 2.5 cm, subdivided into four compartments by disks of 25 cm in diameter (Ugo Basile, model 7600). During the measurements, the bar rotates at a constant speed of 22 rotations per minute. Mice were selected 24 h before the IAS session (during the familiarization phase) by eliminating those that did not remain on the bar for two consecutive periods of 180 s. Selected mice were then evaluated 0.5, 2, 6, 12, 24, 36, and 48 h after the IAS session (Supplementary Figure S1A). The cutoff time used was 180 s. No alteration in the motor function of mice that underwent IAS was observed in comparison with sham animals (Supplementary Figure S1A).

### Spinal Cord Immunofluorescence Analysis Using Confocal Microscopy

Spinal cord immunofluorescence assay was performed 24 h after the swimming session. For this purpose, mice were perfused through the ascending aorta with phosphate buffered saline (PBS) followed by 4% paraformaldehyde (PFA), and L_4_–L_6_ segments of the spinal cord were accurately dissected out and post fixed in 4% PFA for 24 h. After this period, PFA was replaced by a solution of 30% saccharose and incubated for 3 additional days. The spinal cord segments were then properly washed with PBS and embedded in optimum cutting temperature (O.C.T.) using Tissue-Tek^®^ reagent (Sakura^®^ Finetek United States, Torrance, CA, USA). Sections of 10 μm were cut in a cryostat (CM1520, Leica Biosystems, Richmond, IL, USA) and processed for immunofluorescence (four samples per mouse per slide/four animals per group). All the sections were initially blocked with a buffer solution [500 μl per slide containing PBS plus 0.1% Tween 20 plus 5% bovine serum albumin (BSA)] for 2 h at room temperature and subsequently incubated overnight at −4°C with a solution containing primary antibodies against target proteins. Next, a new incubation with secondary antibodies was performed for 1 h at room temperature. For GFAP and Iba-1 primary antibodies, Alexa Fluor 488 secondary antibody was used. For pNFκB primary antibody, immunoglobulin G (IgG)-horseradish peroxidase (HRP) secondary antibody was used. For CX_3_CR1 detection, heterozygous CX_3_CR1-eGPF^+/-^ C57BL/6 mice were used. For c-Fos and NeuN, Alexa Fluor 488 and Alexa Fluor 647 secondary antibodies were used, respectively. The description of product codes, dilutions used, and manufacturers are presented in Supplementary Table S2. To count NeuN/c-Fos double-positive cells, five random fields were counted in each slice, and the average of these five fields per animal was plotted to subsequently conduct the analysis of the mean of the means of the n of 4 mice per group. The analyses were always performed in fields encompassing regions of laminae 1, 2, and 5 of the dorsal horn of the spinal cord. The assembly of the slides was conducted using ProLong^TM^ Gold Antifade Mountant with DAPI melting media (#P36931, Thermo Fisher Scientific, Waltham, MA, USA) or Fluoromount^TM^ Aqueous Mounting Medium (#F4680, Sigma-Aldrich, St. Louis, MO, USA). Analysis using slides with secondary antibodies alone was conducted in parallel as controls to ensure that unspecified staining did not occur. Immunofluorescence analyses were performed in the dorsal horn of the spinal cord in the magnification of ×10 or ×20 as indicated in the figure legends. The images and analyses were performed using a confocal microscope (TSC SP8, Leica Microsystems, Mannheim, Germany).

### Evaluation of Muscle Mechanical Hyperalgesia

Muscle mechanical hyperalgesia was tested in mice 6–48 h after the swimming session as previously reported (Cunha et al., 2004). The test consisted of evoking a hind paw flexion reflex with a handheld force transducer (electronic von Frey aesthesiometer; Insight, Ribeirão Preto, SP, Brazil) adapted with a 0.5-mm^2^ contact area polypropylene tip (Borghi et al., 2014b). During the experiments, only the right limb of animals was evaluated. The applied pressure to hind paw surface induces an articular movement on the ankle joint (dorsiflexion), leading to stretch of the Achilles tendon, which in turn promotes an exaggerated muscle movement response (movement-induced hyperalgesia) when there is sensitization of nociceptors of calf muscle. The end point was characterized by the removal of the paw followed by clear flinching movements. Muscle distension is sufficient to trigger muscle nociceptive responses (Borghi et al., 2014a; Borghi et al., 2014b). After the paw withdrawal, the intensity of the pressure was recorded automatically by the von Frey apparatus. The value of the response was an average of three measurements performed by the experimenter. The results are expressed by delta (Δ) withdrawal threshold (in g) calculated by subtracting the mean measurements (indicated time points) after swimming from the baseline measurements (obtained at rest). The basal mechanical withdrawal threshold was 8.5 ± 0.2 g (mean ± SEM between the groups, 6 mice per group) before IAS session. There was no difference of basal mechanical withdrawal thresholds between groups in the same experiment. Behavioral experiments were conducted always by the same experimenter who was blinded to the experimental groups in an intention to avoid the risk of bias.

### Nuclear Factor κB Activation Assessment by ELISA

Spinal cord samples were collected 24 h after the swimming session and homogenized in ice-cold lysis buffer (Cell Signaling Technology, Beverly, MA, USA). The homogenates were centrifuged (16,000 g × 10 min × 4°C), and the resultant supernatants were used to assess the levels of total and phosphorylated NF-κB p65 subunit by enzyme-linked immunosorbent assay (ELISA) using PathScan kits (Cell Signaling Technology, Beverly, MA, USA) according to the manufacturer’s instructions. The test indicates the proportion between total NFκB and phosphorylated (p)NFκB in analyzed samples. When the ratio is high, a high amount of total NFκB is detected relative to pNFκB, indicating its activation is lessened in samples under analysis. On the other hand, when the total NFκB/pNFκB ratio is low, a greater amount of phosphorylated protein is detected relative to total NFκB, indicating high phosphorylation of NFκB relative to total NFκB in the sample. The results are presented as the sample ratio of NFκB activation (total p65/phospho-p65) per milligram of spinal cord tissue measured at 450 nm (Borghi et al., 2016).

### Reverse Transcription and Quantitative Polymerase Chain Reaction Assay

RT-qPCR was performed as previously described (Borghi et al., 2016). After euthanasia process, mouse spinal cord samples were collected (6–48 h after the IAS session, depending on the experiment) for homogenization in TRIzol™ Reagent (Thermo Fisher Scientific). Subsequently, total RNA was isolated according to the manufacturer’s guideline. The purity of total RNA was measured spectrophotometrically, and the wavelength absorption ratio (260/280) was between 1.8 and 2.0 for all preparations. Reverse transcription of total RNA to cDNA and qPCR were carried out using GoTaq^®^ 2-Step RT-qPCR System (Promega) and target primers. qPCR reaction was performed in Step One Plus TM Real-Time PCR System (Applied Biosystems^®^). The relative gene expression was measured using the comparative 2^-(∆∆Cq)^ method. The primers used in this study are shown in Supplementary Table S3. The expression of *β-actin* mRNA was used as a control for tissue integrity in all samples.

### Quantitation of Tumor Necrosis Factor-α, Interleukin-1β, and Interleukin-10 by ELISA

Spinal cord samples were collected 24 h after the IAS in PBS buffer containing protease inhibitors (500 μl), homogenized, and centrifuged to obtain the supernatant. TNF-α (#DY410), IL-1β (#DY401), and IL-10 (#DY417) production was determined by ELISA using R&D Systems, Inc., kits (Minneapolis, MN, USA). During the assay, plates were coated overnight at 4°C with an immunoaffinity-purified polyclonal antibody specifically for each cytokine. Recombinant murine TNF-α, IL-1β, and IL-10 standards at various dilutions and the samples were added in duplicate and incubated by an additional period (2 h) at room temperature. Rabbit biotinylated immunoaffinity-purified antibodies anti-TNF-α, anti-IL-1β, and anti-IL-10 were added, followed by another incubation at room temperature for 1 h. Finally, avidin-HRP was added to each well, and after 30 min, the plates were washed and the color reagent o-phenylenediamine was added. After blocking reactions, measurements were performed spectrophotometrically at 450 nm (Multiskan GO Microplate Spectrophotometer, Thermo Fisher Scientific, Vantaa, Finland). The results were expressed as picograms (pg) of cytokine per milligram of spinal cord tissue.

### Nitroblue Tetrazolium Reduction Test for Determining the Production of Superoxide Anion

Spinal cord samples were collected 24 h after the swimming session and homogenized with 500 μl of saline solution, and 50 μl of the resultant homogenate was placed in a sterilized 96-well plate, followed by the addition of 100 μl of NBT solution (1 mg/ml) and incubation for 1 h at 37°C. The supernatant was carefully removed from the plates, and the precipitated formazan remaining in the wells was then solubilized by adding 120 μl of 2 M KOH and 140 μl of DMSO. Superoxide anion production was determined spectrophotometrically by the reduction of the redox dye NBT. Readings were performed at 600 nm (Multiskan GO Microplate Spectrophotometer, Thermo Fisher Scientific, Vantaa, Finland). The results were presented as NBT reduction through optical density measurements of tissue samples (OD/mg of spinal cord) (Borghi et al., 2016).

### Lipid Peroxidation Assay

Lipid peroxidation levels induced by IAS session in the spinal cord samples were assessed by determining thiobarbituric acid reactive substances (TBARS) levels using an adapted method previously described (Borghi et al., 2016). Mice were euthanized 24 h after the swimming session, and samples of spinal cord were collected for lipid peroxidation assessment. Malondialdehyde (MDA), an intermediate product of lipid peroxidation, was determined by the difference between two absorbances performed at 535 and 572 nm using a microplate spectrophotometer reader (Multiskan GO Microplate Spectrophotometer, Thermo Fisher Scientific, Vantaa, Finland). The results were presented as lipid peroxidation (nmol of MDA/mg of tissue).

### Statistical Analysis

Results are presented as means ± SEM of measurements made on 4–6 mice in each group per experiment, depending on the experiment, and are representative of two separate experiments. Two-way analysis of variance (ANOVA) was used to compare the groups and doses at all time points (curves). The analyzed factors were treatments, time, and time vs. treatment interaction. When there was a significant time vs. treatment interaction, one-way ANOVA followed by Tukey’s t-test was performed for each time. Statistical differences were significant at p < 0.05.

## Results

### Intense Acute Swimming Induced the Activation of Spinal Cord Astrocytes and Microglia After 24 h

**FIGURE 2:**
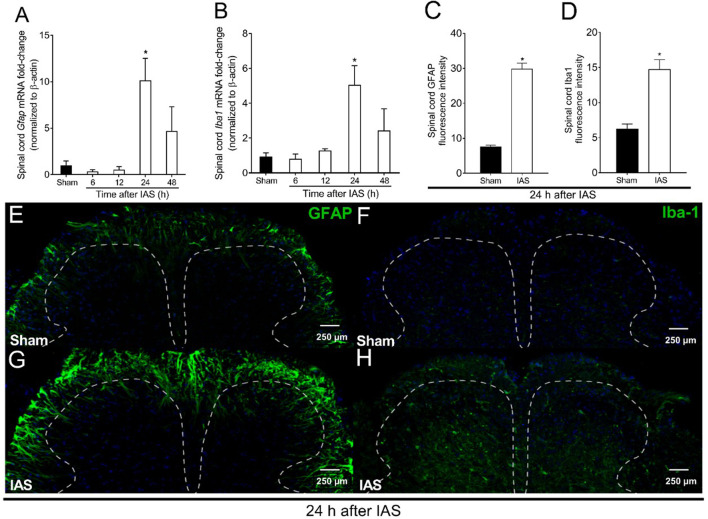
Unaccustomed IAS session induces spinal cord astrocyte and microglial activation in the spinal cord dorsal horn. **(A)** *Gfap* and (**B)** *Iba1* mRNA expression was determined in sham and exercised mice 6–48 h after the intense acute swimming session by RT-qPCR. At time 24 h after the exercise session (peak of *Gfap* and *Iba1* mRNA expression), immunofluorescence analysis of the spinal cord samples was performed to confirm **(C)** GFAP and **(D)** Iba-1 expression (the percentage of GFAP and Iba-1 fluorescence intensity in each experimental group). Spinal cord samples were stained with antibodies for astrocytes **(E, G)** and microglia **(F, H)** (GFAP and Iba-1, respectively; green) and nucleus (DAPI, blue) detection. Representative immunostainings of the spinal cord of sham and exercised mice are shown in panels **E–H** (×20 magnification, scale bar 250 μm). Results are presented as spinal cord *gfap and Iba1* mRNA expression fold change (normalized to β-actin) (*n* = 6 mice per group per experiment, representative of two independent experiments) and as spinal cord GFAP and Iba-1 fluorescence intensity (*n* = 4 mice per group per experiment, representative of two independent experiments). *p < 0.05 compared to sham mice (one-way ANOVA followed by Tukey’s posttest).

In the first set of experiments, we evaluate the time-course response of spinal cord *Gfap* and *Iba1* mRNA expression as indicative of astrocyte and microglial activation, respectively. The time points were between 6 and 48 h after the mice experienced unaccustomed IAS session (Figure 2). No increase in the mRNA expression of *Gfap* and *Iba1* was detected in comparison to that in the sham mice at 6 and 12 h post-exercise session. On the other hand, the IAS group presented a significant increase of *Gfap* and *Iba1* mRNA expression compared to that in the sham group at the 24th hour (Figures 2A, B). To validate the mRNA expression data at the protein level, immunofluorescence assay was conducted 24 h after the IAS session. The activation of spinal cord astrocytes and microglia was confirmed bilaterally in IAS but not in sham mice (Figures 2C–H). Based on previous studies of our laboratory (Borghi et al., 2014a; Borghi et al., 2014b; Borghi et al., 2015; Borghi et al., 2016), mechanical hyperalgesia starts to increase 6 h after the unaccustomed IAS session and reaches its peak at 24 h after the exercise, which according to the present data coincides with the activation of astrocytes and microglia in the spinal cord.

### Treatments With Glial Inhibitors by Intrathecal Route Diminished Intense Acute Swimming-Induced Mechanical Hyperalgesia

**FIGURE 3:**
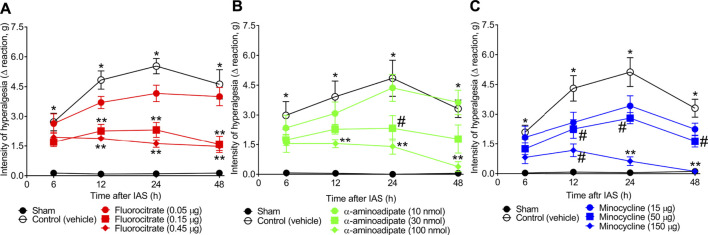
**(A)** Fluorocitrate, **(B)** α-aminoadipate, and **(C)** minocycline reduce, in a dose-dependent manner, IAS-induced muscle mechanical hyperalgesia. Mice received vehicle (saline, i.t.), fluorocitrate (0.05–0.45 µg, i.t.), α-aminoadipate (10–1,000 nmol, i.t.), or minocycline (15–150 μg, i.t.) 30 min before experiencing unaccustomed intense acute swimming session. The intensity of muscle mechanical hyperalgesia was evaluated 6–48 h after the exercise session. Results are presented as intensity of hyperalgesia (Δ reaction, in grams; *n* = 6 mice per group per experiment and is representative of two independent experiments). *p < 0.05 compared with sham group, #p < 0.05 compared with vehicle group, **p < 0.05 compared with vehicle and with the lowest dose tested of the compound (two-way ANOVA followed by Tukey’s *post-hoc*).

To evaluate the role of spinal cord astrocytes and microglia in unaccustomed IAS-induced muscle pain, animals received a single local (i.t.) treatment with vehicle or well-known glial inhibitors fluorocitrate (0.05–0.45 µg; astrocyte inhibitor), α-aminoadipate (10–100 nmol; astrocyte inhibitor), and minocycline (15–150 μg; microglial inhibitor) 30 min before the exercise session. Mechanical hyperalgesia was evaluated from 6 to 48 h after the swimming (Figure 3). Fluorocitrate, α-aminoadipate, and minocycline i.t. treatments dose-dependently reduced muscle mechanical hyperalgesia induced by IAS session from the 12th hour onward, with the highest doses of each compound being the most effective in inhibiting unaccustomed IAS-induced pain, except for the intermediate dose of fluorocitrate that achieved a similar effect as the higher dose (Figures 3A–C). In the case of α-aminoadipate and minocycline, they abolished IAS-induced mechanical hyperalgesia at 48 h after the exercise session (Figures 3B, C). Thus, the intermediate dose of fluorocitrate and the higher doses of α-aminoadipate and minocycline were selected for the next set of experiments. These results corroborate the notion that spinal cord astrocyte and microglia activation is necessary to induce DOMS, especially between 24 and 48 h post-exercise (time of hyperalgesia peak).

### Spinal Cord Astrocytes and Microglia Mediate Intense Acute Swimming-Induced Mechanical Hyperalgesia in a Nuclear Factor κB-Dependent Manner

**FIGURE 4:**
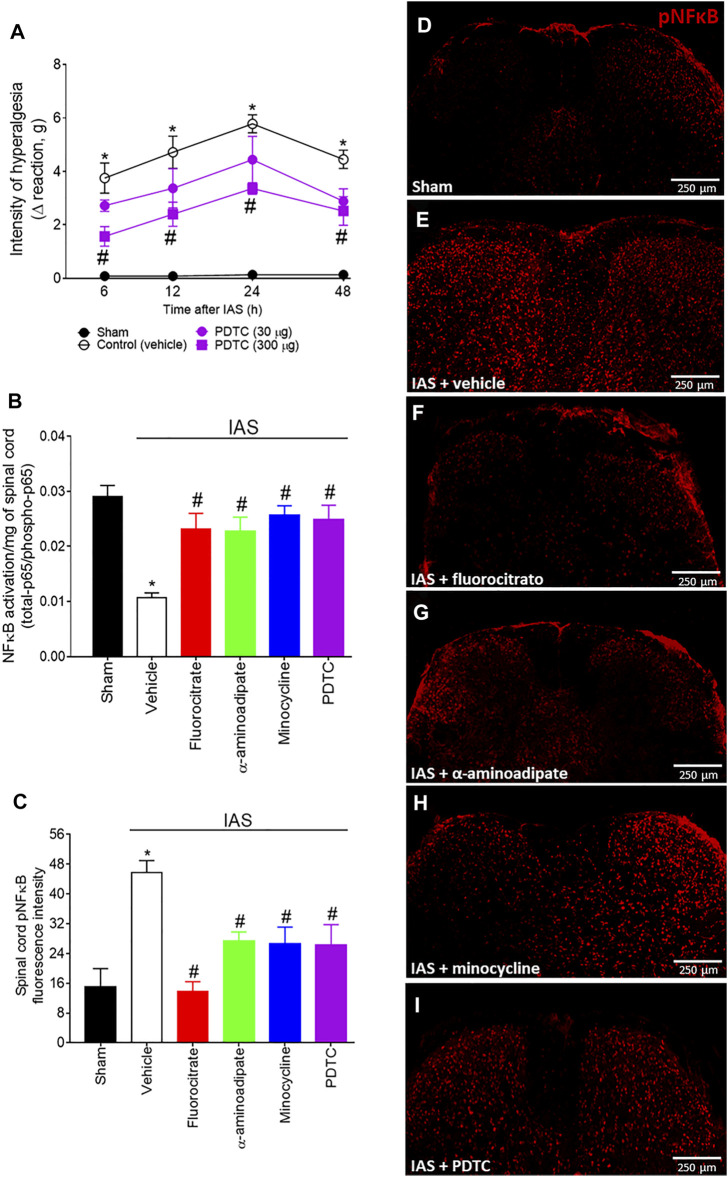
**(A)** PDTC reduces in a dose-dependent manner IAS-induced muscle mechanical hyperalgesia. Mice received vehicle (saline, i.t.) and PDTC (30–300 μg, i.t.) 30 min before experiencing unaccustomed intense acute swimming session. The intensity of muscle mechanical hyperalgesia was evaluated 6–48 h after the exercise session. Results are presented as intensity of hyperalgesia (Δ reaction, in grams; *n* = 6 mice per group per experiment and is representative of two independent experiments). *p < 0.05 compared to the sham group, #p < 0.05 compared to the vehicle-treated group (two-way ANOVA followed by Tukey’s *post-hoc*). Fluorocitrate (0.15 µg, i.t.), α-aminoadipate (1,000 nmol, i.t.), minocycline (150 μg, i.t.), and PDTC (300 μg, i.t.) inhibit **(B)** spinal cord NFκB activation (total NFκB/phosphorylated NFκB ratio) and **(C–I)** pNFκB immunoreactivity (the percentage of pNFκB fluorescence intensity in each experimental group) 24 h after the exercise session. Control mice received saline i.t. as vehicle. Representative immunostainings of the spinal cord of sham and exercised-treated mice are shown in panels **D–I** (×10 magnification, scale bar 250 μm). Results are presented as NFκB activation (total-p65/phosphotrilated-p65 ratio)/mg of spinal cord (*n* = 6 mice per group per experiment, representative of two independent experiments) and as spinal cord pNFκB fluorescence intensity, respectively (*n* = 4 mice per group per experiment, representative of two independent experiments). *p < 0.05 compared to the sham group, #p < 0.05 compared to the vehicle-treated group (one-way ANOVA followed by Tukey’s *post-hoc*).

Our next goal was to evaluate whether spinal cord NFκB has a role in mediating IAS-induced DOMS (Figure 4). In our first approach, mice received a single i.t. treatment with vehicle or the selective NFκB inhibitor PDTC (30–300 µg) 30 min before the exercise session, and mechanical hyperalgesia was evaluated from 6 to 48 h after IAS (Figure 4A). PDTC dose-dependently reduced IAS-induced muscle mechanical hyperalgesia, with significant inhibition from 6 to 48 h with the dose of 300 µg (Figure 4A). Subsequently, the efforts were focused on verifying the relationship between spinal cord glial cells and NFκB in the peak of IAS-induced DOMS (24 h after the exercise session) (Figures 4B–I). For this purpose, spinal cord NFκB activation was evaluated in mice treated by i.t. route with fluorocitrate, α-aminoadipate, minocycline, and PDTC (control drug) using ELISA (Figure 4B) and immunofluorescence (Figures 4C–I) approaches. Spinal cord NFκB activation was triggered by IAS [decreased the ratio of total NFκB/pNFκB (the activated form is the phosphorylated one)], which was reversed by the treatments with all tested glial inhibitors (Figure 4B). PDTC i.t. treatment also reverted NFκB activation in the spinal cord of IAS animals, further corroborating that its effect on hyperalgesia was due to the inhibition of spinal cord NFκB activation (Figure 4B). Using immunofluorescence analysis, we demonstrate that pNFκB staining in spinal cord dorsal horn is elevated at 24 h in IAS animals compared to sham animals (Figures 4C–E). In agreement with ELISA data (Figure 4B), spinal cord pNFκB staining was reduced upon i.t. treatment with glial and NFκB inhibitors (Figures 4F–I). These results suggest the participation of spinal cord NFκB and spinal cord glial cell in NFκB activation during DOMS.

### Spinal Cord CX_3_CL1/CX_3_CR1 Signaling Contribution to Intense Acute Swimming-Induced Mechanical Hyperalgesia

**FIGURE 5:**
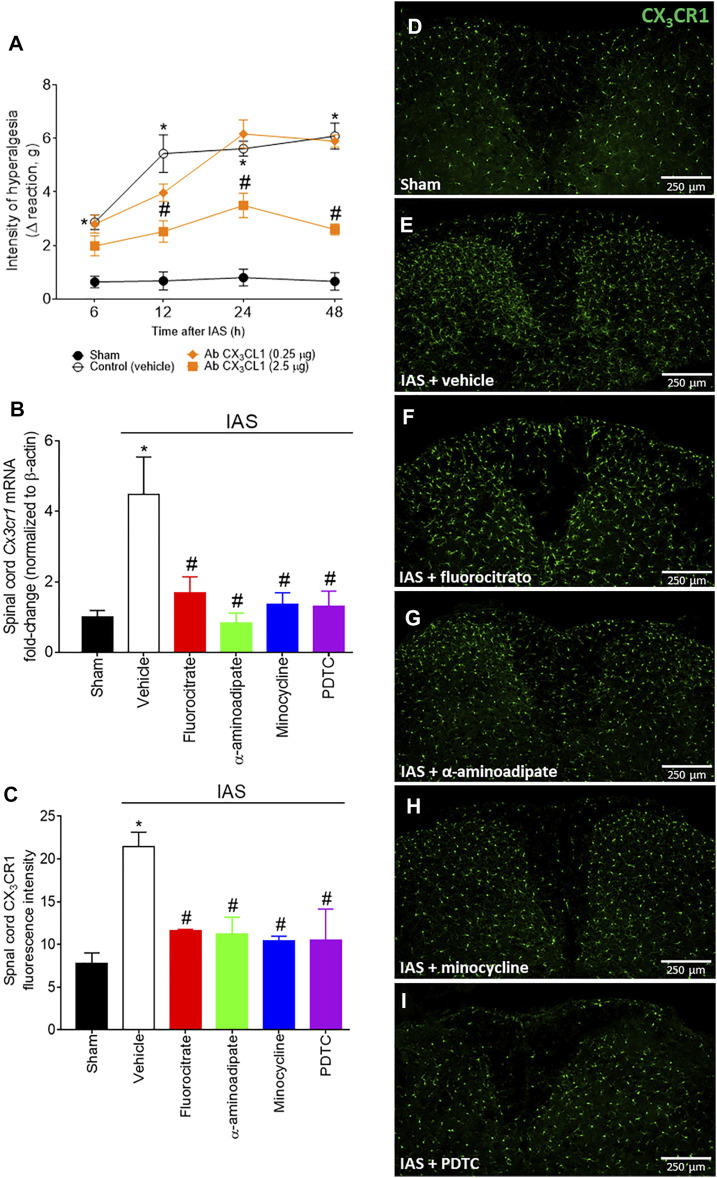
**(A)** Antibody anti-CX_3_CL1 reduces IAS-induced muscle mechanical hyperalgesia. Mice received vehicle (IgG, i.t.) and Ab CX_3_CL1 (0.25–2.5 µg, i.t.) 30 min before experiencing unaccustomed intense acute swimming session. The intensity of muscle mechanical hyperalgesia was evaluated 6–48 h after the exercise session. Results are presented as intensity of hyperalgesia (Δ reaction, in grams; *n* = 6 mice per group per experiment and is representative of two independent experiments). *p < 0.05 compared to the sham group, #p < 0.05 compared to the vehicle-treated group (two-way ANOVA followed by Tukey’s *post-hoc*). Fluorocitrate (0.15 µg, i.t.), α-aminoadipate (1,000 nmol, i.t.), minocycline (150 μg, i.t.), and PDTC (300 μg, i.t.) inhibit **(B)** spinal cord *Cx3cr1* expression and **(C–I)** CX_3_CR1 reporter fluorescence (the percentage of CX_3_CR1 fluorescence intensity in each experimental group) 24 h after the exercise session. Control mice received saline i.t. as vehicle. Representative immunostainings of the spinal cord of sham and exercised-treated mice are shown in panels **D–I** (×10 magnification, scale bar 250 μm). Results are presented as spinal cord *Cx3cr1* mRNA expression fold change (normalized to β-actin) (*n* = 6 mice per group per experiment, representative of two independent experiments) and as spinal cord CX_3_CR1 reporter fluorescence intensity, respectively (*n* = 4 mice per group per experiment, representative of two independent experiments). *p < 0.05 compared to the sham group, #p < 0.05 compared to the vehicle-treated group (one-way ANOVA followed by Tukey’s *post-hoc*).

CX_3_CL1 is released by primary afferent neurons in the spinal cord and activates its receptor CX_3_CR1, expressed mainly by microglia. This intercellular event represents a potential spinal cord mechanism during central sensitization, evidencing how peripheral neurons activate microglia in the spinal cord (Pinho-Ribeiro et al., 2017). The participation of CX_3_CL1/CX_3_CR1 signaling would sum up to corroborate the participation of spinal cord glial cells in DOMS (Figure 5). Mice received a single i.t. treatment of isotype control IgG antibody or neutralizing antibody anti-CX_3_CL1 (Ab CX_3_CL1; 0.25–2.5 µg) 30 min before the exercise session, and mechanical hyperalgesia was evaluated from 6 to 48 h after swimming (Figure 5A). The lowest dose of the Ab CX_3_CL1 had no effect on mechanical hyperalgesia; however, 2.5 µg dose efficiently inhibited mechanical hyperalgesia from 12 h onward (Figure 5A). Further extending on this topic, we next evaluated the mRNA expression of spinal cord *Cx3cr1* 24 h after IAS (peak of DOMS) and if inhibiting glial cells and NFκB at this time point influences its mRNA expression (Figure 5B). Unaccustomed IAS significantly enhanced *Cx3cr1* mRNA expression in comparison to sham group, while i.t. treatments with fluorocitrate, α-aminoadipate, minocycline, and PDTC inhibited its expression (Figure 5B). Corroborating the mRNA expression data, IAS CX_3_CR1-eGFP^+/-^ mice (24 h) presented higher fluorescence intensity than sham CX_3_CR1-eGFP^+/-^ mice, indicating higher CX_3_CR1 protein levels in IAS animals than in sham animals. The i.t. treatment with glial and NFκB inhibitors reduced CX_3_CR1 fluorescence in IAS animals (Figures 5C–I). These data show that spinal cord CX_3_CL1/CX_3_CR1 signaling plays a role in DOMS and that the expression of this chemokine receptor is regulated by glial cells and NFκB in the spinal cord during DOMS.

### Targeting Spinal Cord Tumor Necrosis Factor-α and Interleukin-1β and Exogenous Interleukin-10 Intrathecal Administration Inhibit Intense Acute Swimming-Induced Mechanical Hyperalgesia

**FIGURE 6:**
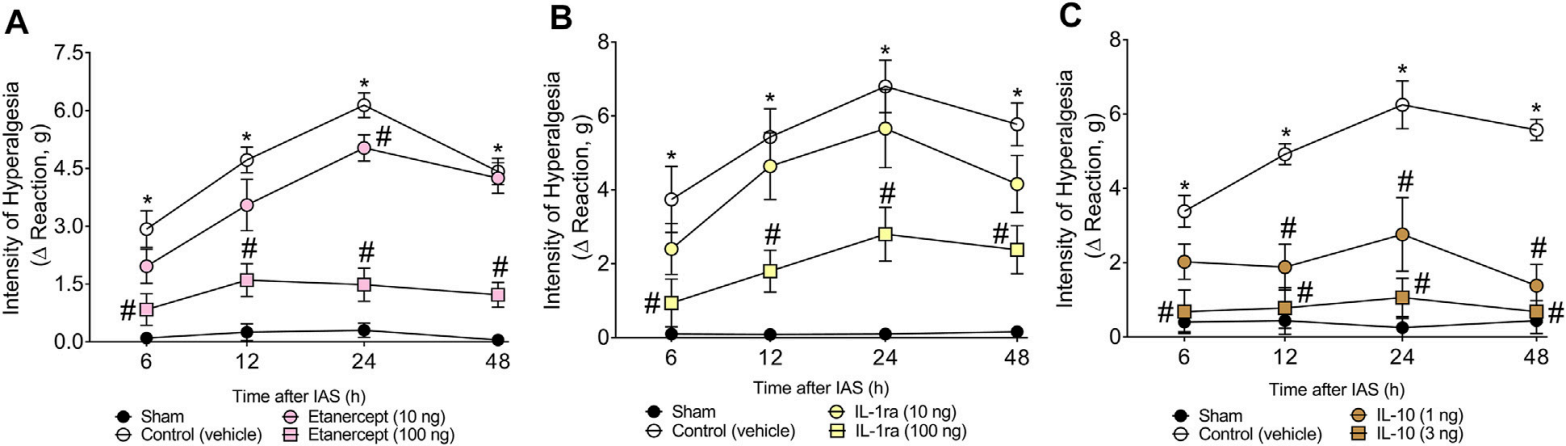
**(A)** Etanercept, **(B)** IL-1ra, and **(C)** mRIL-10 reduce IAS-induced muscle mechanical hyperalgesia. Mice received vehicle (saline, i.t.), etanercept (10–100 ng, i.t.), IL-1ra (10–100 ng, i.t.), and mRIL-10 (1–3 ng, i.t.) 30 min before experiencing unaccustomed intense acute swimming session. The intensity of muscle mechanical hyperalgesia was evaluated 6–48 h after the exercise session. Results are presented as intensity of hyperalgesia (Δ reaction, in grams; *n* = 6 mice per group per experiment and is representative of two independent experiments). *p < 0.05 compared with sham group, #p < 0.05 compared with vehicle group (two-way ANOVA followed by Tukey’s *post-hoc*).

After determining the role of spinal cord astrocytes, microglia, NFκB, and CX_3_CL1/CX_3_CR1 signaling in unaccustomed IAS-induced DOMS, the participation of spinal cord cytokines TNF-α, IL-1β, and IL-10 was evaluated using immune-pharmacological tools. For this purpose, mice received a single i.t. treatment of vehicle or TNF-α blocker etanercept (10–100 ng), IL-1 receptor antagonist (IL-1ra), or mouse recombinant IL-10 (1–3 ng) (Figure 6). The dose of 10 ng of etanercept only inhibited mechanical hyperalgesia at 24 h; however, the dose of 100 ng was effective in inhibiting mechanical hyperalgesia between 6 and 48 h (Figure 6A). Regarding IL-1ra, the dose of 10 ng was ineffective, whereas the dose of 100 ng inhibited mechanical hyperalgesia between 6 and 48 h (Figure 6B). Finally, i.t. treatment with 1 ng of IL-10 inhibited mechanical hyperalgesia in the 12th and 24th hour after the exercise session, whereas the dose of 3 ng abolished mechanical hyperalgesia at all evaluated times (Figure 6C). These results point to the participation of spinal cord TNF-α and IL-1β in mediating IAS-induced DOMS, while IL-10 is important to counteract it.

### Targeting Spinal Cord Astrocytes, Microglia, and Nuclear Factor κB Inhibits Intense Acute Swimming-Induced *Tnfα*, *Il1β*, and *Il10* mRNA Expression and Protein Levels

**FIGURE 7:**
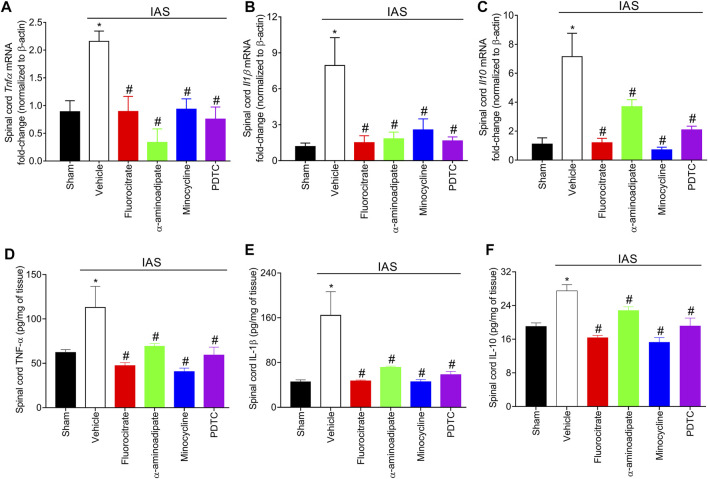
Fluorocitrate (0.15 µg, i.t.), α-aminoadipate (1,000 nmol, i.t.), minocycline (150 μg, i.t.), and PDTC (300 μg, i.t.) inhibit spinal cord **(A)** *Tnfα*, **(B)** *Il1β*, and **(C)** *Il10* mRNA expression and **(D)** TNF-α, **(E)** IL-1β, and **(F)** IL-10 protein levels 24 h after IAS session. Control mice received saline i.t. as vehicle. Results are presented as spinal cord *Tnfα*, *Il1β*, and *Il10* mRNA expression fold change (normalized to β-actin) and as spinal cord TNF-α, IL-1β, and IL-10 in pg/ml, respectively (*n* = 6 mice per group per experiment and is representative of two independent experiments). *p < 0.05 compared to the sham group, #p < 0.05 compared to the vehicle-treated group (one-way ANOVA followed by Tukey’s *post-hoc*).

Considering that cytokines (Figure 6) participate in unaccustomed IAS-induced DOMS, we next quantitated the mRNA expression and protein levels of TNF-α, IL-1β, and IL-10 in the spinal cord after inhibition of glial cells and NFκB (Figure 7). For this investigation, in the first set of experiments, mice received a single i.t. treatment of vehicle, fluorocitrate, α-aminoadipate, minocycline, or PDTC 30 min before IAS session. The mRNA expression of cytokines was evaluated at the peak DOMS (24 h). The increased mRNA expression of *Tnfα*, *Il1β*, and *Il10* induced by IAS was successfully inhibited by i.t. treatments with glial and NFκB inhibitors (Figures 7A–C). Spinal cord TNF-α, IL-1β, and IL-10 protein levels were also increased after IAS compared to sham animals in the peak of DOMS (24 h), while i.t. treatment with glial and NFκB inhibitors significantly reduced IAS-induced cytokine production (Figures 7D, E). These data demonstrate that spinal cord astrocytes, microglia, and NFκB activation mediates neuroinflammation in DOMS, as well as their inhibition counteracts the expression of classical cytokines that have pro-hyperalgesic or anti-hyperalgesic roles.

### Targeting Spinal Cord Astrocytes, Microglia, and Nuclear Factor κB Inhibits Intense Acute Swimming-Induced Oxidative Stress

**FIGURE 8:**
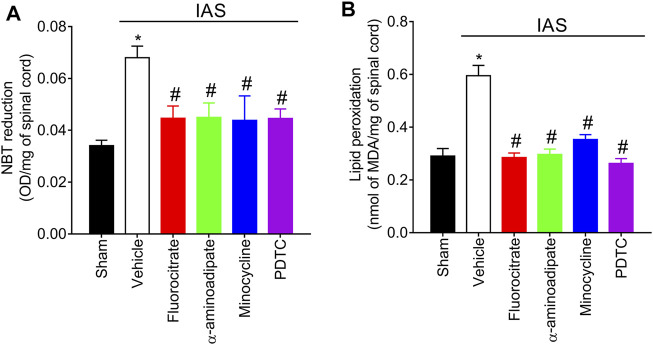
Fluorocitrate (0.15 µg, i.t.), α-aminoadipate (1,000 nmol, i.t.), minocycline (150 μg, i.t.), and PDTC (300 μg, i.t.) inhibit spinal cord **(A)** superoxide anion production and **(B)** lipid peroxidation levels 24 h after IAS session. Control mice received saline i.t. as vehicle. Results are presented as spinal cord NBT reduction (OD/mg of spinal cord) and lipid peroxidation (nmol of MDA/mg of spinal cord), respectively (*n* = 6 mice per group per experiment and is representative of two independent experiments). *p < 0.05 compared to the sham group, #p < 0.05 compared to the vehicle-treated group (one-way ANOVA followed by Tukey’s *post-hoc*).

Evidence supports that IAS induces oxidative stress in the spinal cord and that this redox imbalance also accounts for IAS-induced hyperalgesia and NFκB activation (Borghi et al., 2016). Mice were treated with a single i.t. administration of vehicle, fluorocitrate, α-aminoadipate, minocycline, or PDTC 30 min before the swimming session, and the levels of spinal cord superoxide anion (NBT reduction assay) and lipid peroxidation (MDA concentration assay) were assessed at the peak of DOMS (24 h) (Figure 8). Unaccustomed IAS session significantly elevated the levels of spinal cord superoxide anion and lipid peroxidation compared to sham mice. I.t. treatments with glial and NFκB inhibitors reduced the production of superoxide anion and lipid peroxidation (Figures 8A, B). This evidence demonstrates that inhibition of spinal cord astrocytes, microglia, and NFκB also as a consequence diminishes oxidative stress.

### Intense Acute Swimming Induces Neuronal Activation in the Spinal Cord, Which Is Amenable by Glial and Nuclear Factor κB Inhibitors

**FIGURE 9:**
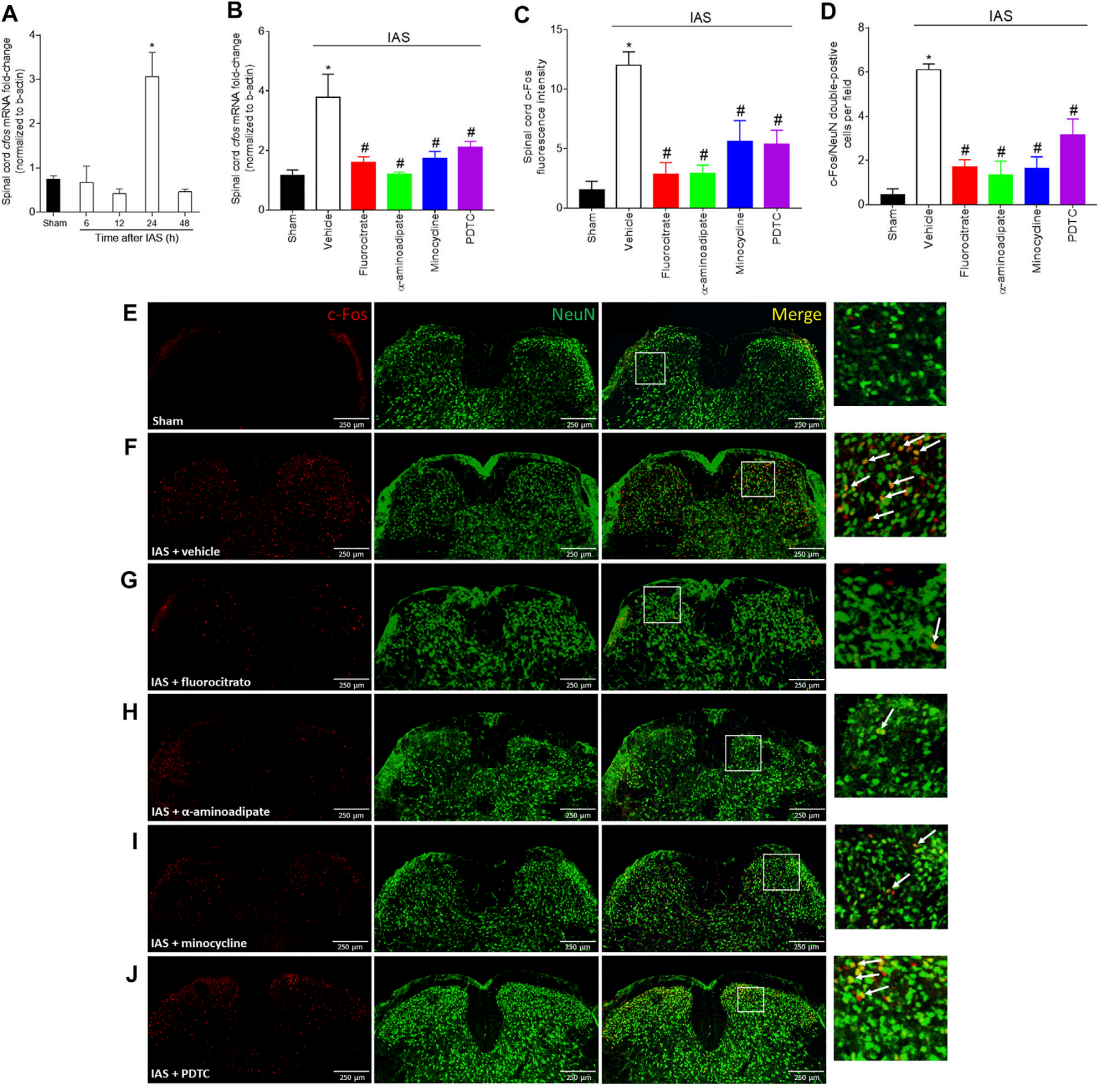
Unaccustomed IAS session induces spinal cord dorsal horn c-Fos activation. **(A)** The *cfos* mRNA expression was determined in sham and exercised mice 6–48 h after the intense acute swimming session by RT-qPCR. At time 24 h after the exercise session (peak of *cfos* mRNA expression), RT-qPCR and immunofluorescence analysis of the spinal cord samples were performed under vehicle (5 μl, i.t.), fluorocitrate (0.15 µg, i.t.), α-aminoadipate (1,000 nmol, i.t.), minocycline (150 μg, i.t.), and PDTC (300 μg, i.t.) treatments to evaluate **(B)** *cfos* mRNA expression and **(C–J)** c-Fos protein immunodetection. Panel **C** presents the percentage of c-Fos fluorescence intensity in each experimental group. **(D)** Mean of quantification of double-positive cells per experimental group. **(E–J)** Spinal cord samples were double-stained with antibodies for c-Fos (neuronal activation; red) and NeuN (neuron nuclear marker; green) detection. Representative immunostainings of the spinal cord of sham and exercise-treated mice are shown in panels **D–I** (×10 magnification, scale bar 250 μm); zoom in inset panels on right; white arrows denote c-Fos/NeuN double-positive cells. Results are presented as spinal cord *cfos* mRNA expression fold change (normalized to β-actin) (*n* = 6 mice per group per experiment, representative of two independent experiments), as spinal cord c-Fos fluorescence intensity, and as c-Fos/NeuN double-positive cells per field, respectively (*n* = 4 mice per group per experiment, representative of two independent experiments). *p < 0.05 compared to sham mice, #p < 0.05 compared to the vehicle-treated group (one-way ANOVA followed by Tukey’s posttest).

To further explore the mechanisms underlying neuroinflammation in DOMS, we next investigated spinal cord neuronal activation in IAS through c-Fos evaluation and whether the activity of glial cells and NFκB would influence the neuronal activation in the spinal cord (Figure 9). In the first round of experiments, the time-course response of spinal cord *cfos* mRNA expression from 6 to 48 h after IAS session was evaluated (Figure 9A). In the first 12 h, no increase in the expression of *cfos* was detected; however, a sharp and significant increase in its expression was observed at 24 h after IAS (Figure 9A). Thus, 24 h was selected as the time point of analyses in the following experiments. The next investigation was to determine whether inhibiting glia and NFκB would alter the increase of *cfos* mRNA expression. Mice were treated once by i.t. route with vehicle, fluorocitrate, α-aminoadipate, minocycline, or PDTC 30 min before IAS session, and after 24 h, the mRNA expression of *cfos* was evaluated. The IAS-induced *cfos* mRNA expression was inhibited by i.t. treatments with glial and NFκB inhibitors (Figure 9B). In the next step, mice were treated with vehicle, fluorocitrate, α-aminoadipate, minocycline, or PDTC 30 min before IAS session, and c-Fos staining was analyzed in spinal cord samples collected at 24 h (Figures 9C–J). The immunodetection of c-Fos was increased in IAS animals compared to sham group, while i.t. treatments with glial and NFκB inhibitors significantly reduced c-Fos detection (Figures 9C–J). We also used NeuN as a neuronal marker to identify neurons in the spinal cord dorsal horn that were positive for c-Fos. The number of c-Fos/NeuN double-positive cells was significantly higher in IAS animals than in sham animals (Figures 9D–J). Inset panels in the right side (Figures 9E–J) and quantitation of c-Fos/NeuN double-positive cells (Figure 9D) showed that i.t. treatments with glial and NFκB inhibitors reduced the presence of these double-positive cells in the spinal cord dorsal horn. These results suggest the participation of spinal cord astrocytes, microglia, and NFκB in the modulation of spinal cord neuronal activation during DOMS.

## Discussion

The present study demonstrates that DOMS caused by unaccustomed intense acute aerobic swimming exercise (IAS) involves neuroinflammatory events in the spinal cord. The primary afferent neurons contribute to this neuroinflammatory process by releasing CX_3_CL1 in the spinal cord and activating microglia, thus initiating a neuroimmune communication that produces cytokines and oxidative stress and activates NFκB, causing the sensitization of nociceptor sensory neurons in the spinal cord, which is observed as DOMS.

DOMS may emerge after both acute high-intensity and prolonged exercise sessions (MacIntyre et al., 1995; Cheung et al., 2003; Dannecker and Koltyn, 2014; Dos Santos et al., 2020). In humans, evidence demonstrates that eccentric exercise modulates spinal sites in DOMS, accounting for central sensitization (Hosseinzadeh et al., 2013). The study applied nociceptive withdrawal reflex test to measure sensitivity in the spinal cord system and found a reduction of approximately 30% of nociceptive withdrawal reflex threshold on the first day after the eccentric exercise bout (Hosseinzadeh et al., 2013). Preclinically, few articles demonstrated spinal cord glial reactivity and the role of cytokines using DOMS models (Pereira et al., 2015; Dos Santos et al., 2020). In trained (8 weeks, 5 days per week) mice exposed to a protocol of downhill running, spinal cord dorsal horn astrocytic and microglial activation markers were increased 36 h after the end of the last exercise session in week 8 (Pereira et al., 2015). In a different protocol, mice that performed only one session of running exercise (40 min with progressive speed over the period) presented increased spinal cord dorsal horn microglial activation associated with elevated TNF-α and IL-6 levels in spinal cord 24–48 h after the exercise session. Furthermore, 70 kDa Hsp70, which is a molecule produced upon exercise, activates Toll-like receptor 4 (TLR4) expressed by spinal cord microglia, accounting to explain DOMS (Dos Santos et al., 2020). Our study demonstrates for the first time the involvement of spinal cord dorsal horn glial cells in increased neuronal activity after acute prolonged swimming exercise (120 min) that presents different environmental and physiological aspects in comparison to other aerobic modalities. For example, swimming exercise is characterized by reduced mechanical impact and gravity interference compared to exercise performed out of water. We demonstrate herein that DOMS is a notorious outcome also in this condition and is related to glial activation and function in the spinal cord.

Importantly, our study evaluated the temporal expression of classical markers of astrocytes and microglial activation in the spinal cord after IAS-induced DOMS. Astrocytic (GFAP) and microglial (Iba-1) activation became statistically detectable 24 h after the swimming session at the mRNA and protein levels. To establish a causal relationship between spinal cord gliosis and pain in DOMS, we performed pharmacological approaches using fluorocitrate and α-aminoadipate (inhibitors of astrocyte metabolism) (Voss et al., 2016) and minocycline (selective inhibitor of M1 microglia) (Kobayashi et al., 2013) i.t. treatments. All treatments efficiently inhibited IAS-induced mechanical hyperalgesia in post-recovery exercise period from 12 to 48 h. The absence of mechanical hyperalgesia inhibition by i.t. treatments at early periods (6 h) after exercise is reasonable, since there was no massive activation of glia at least until 24 h.

Next, we demonstrated that glial inhibitors target spinal cord p65 NFκB activation (as demonstrated by ELISA and immunofluorescence analyses) and, additionally, glial and NFκB inhibitors target spinal cord CX_3_CR1 (mRNA expression and CX_3_CR1 reporter mouse), both 24 h after IAS session. A functional experiment using the i.t. treatment of neutralizing antibody anti-CX_3_CL1 demonstrated that inhibiting this chemokine reduces IAS-induced DOMS. NFκB activation is responsible for neuroinflammatory and nociceptive pathologic events in the spinal cord (Zarpelon et al., 2016; Pinho-Ribeiro et al., 2017; Li et al., 2020). Here, i.t. treatment with the NFκB inhibitor PDTC reduced DOMS from 6 to 48 h after the swimming exercise, and i.t. treatment with glial inhibitors reduced NFκB activation, thus suggesting a crucial role of spinal cord glial cells and NFκB activity for DOMS development. Peripheral nociceptor neurons contribute to neuroinflammation and neuronal sensitization by releasing CX_3_CL1 in the spinal cord, which activates CX_3_CR1 in microglia (Milligan et al., 2004; Gao and Ji, 2010). In the periphery, CX_3_CL1 (from muscle-related endothelium) levels are enhanced at mRNA and protein levels in skeletal muscle after one bout of 1 h of aerobic exercise in humans, accounting to macrophage recruitment and inflammatory products (Stromberg et al., 2016). However, to our knowledge, this is the first demonstration of the contribution of spinal cord CX_3_CL1/CX_3_CR1 signaling to IAS-induced DOMS.

IAS-induced DOMS was inhibited 6–48 h after the session when endogenous TNF-α and IL-1β were targeted, just like when exogenous IL-10 was administered. Further extending on this topic, we next evaluate whether IAS modifies the expression of these cytokines in the spinal cord at the peak of DOMS (24 h). IAS enhanced the mRNA expression of *Tnf-α*, *Il-1β*, and *Il-10* as well as their respective protein levels, which were diminished by glial and NFκB inhibitors. NFκB activation enhances TNF-α and IL-1β production (Pahl, 1999); thus, its inhibition reduces the transcription of these genes. The crosstalk between non-neuronal cells using cytokines as mediators has been demonstrated to be essential for central nervous system (CNS) pathology (Liu et al., 2011; Matejuk and Ransohoff, 2020). Spinal cord microglia mediate the activation of resident astrocytes and oligodendrocytes. Microglia release TNF-α and IL-1β, and in turn, TNF-α activates astrocytes (Pinho-Ribeiro et al., 2017). Activated astrocytic networks may as well facilitate microglial activation *via* calcium wave-related adenosine triphosphate (ATP) release, which interacts with its purinergic receptor in microglia and activates these cells (Liu et al., 2011). These concomitant glial-to-glial mechanisms converge to promote spinal cord neuronal sensitization. These mechanisms may explain, for example, the inhibition of IAS-induced spinal cord *Cx3cr1* mRNA expression and its protein levels by fluorocitrate and α-aminoadipate.

IL-10 has been proposed as a crucial cytokine for the control of central sensitization (Milligan et al., 2012; Vanderwall and Milligan, 2019). Here, we observed that i.t. treatment with mrIL-10 abolished DOMS (3 ng dose) 6–48 h after the exercise session. Moreover, the increased *Il10* mRNA expression and protein levels after DOMS (24 h) was reversed by i.t. treatments with glial and NFκB inhibitors. This reduction in IL-10 production instead of an increase during treatments might be a result of diminished pro-hyperalgesic cytokine production, waiving the need of endogenous IL-10 production to limit hyperalgesia. This is supported by evidence demonstrating that endogenous IL-10 limits hyperalgesia by reducing pro-hyperalgesic cytokine production in models of inflammatory pain, neuropathic pain, and IAS-induced DOMS (Poole et al., 1995; Borghi et al., 2015; Krukowski et al., 2016; Vanderwall and Milligan, 2019).

Our study also demonstrated that IAS enhanced the levels of oxidative stress in the spinal cord at the peak of DOMS, as reflected by increased superoxide anion production and lipid peroxidation levels. Astrocytes and microglia can produce large quantities of reactive oxygen species (ROS) in pathological conditions (Wolf et al., 2017; Chen et al., 2020). NFκB activation by ROS also regulates the expression of gp91^phox^ in nicotinamide adenine dinucleotide phosphate (NADPH) oxidase complex, thus contributing to oxidative metabolism (Anrather et al., 2006). Additionally, oxidative stress at the level of spinal cord contributes to inflammatory pain (Wang et al., 2004). In accordance with the abovementioned, we showed that treating mice by i.t. route with glial and NFκB inhibitors reduced IAS-induced oxidative stress and pain, evidencing a role for astrocytes, microglia, and NFκB in DOMS. Further corroborating the contribution of oxidative stress to DOMS, evidence supports that antioxidant treatment reduces IAS-induced DOMS (Borghi et al., 2016).

Finally, an upregulation of the proto-oncogene *cfos* and its nuclear protein c-Fos in the spinal cord dorsal horn neurons (NeuN-positive cells) of IAS animals was observed at the peak of mechanical hyperalgesia (24 h). c-Fos has been broadly used as a marker of nociceptive neuron activity in the superficial laminae of spinal cord dorsal horn following peripheral stimulatory events (Hunt et al., 1987; Bullitt, 1990). In IAS animals, there was increased immunodetection of c-Fos in the region of superficial laminae where the axons of primary afferent neurons enter the spinal cord. Thus, further confirming that peripheral inflammatory events triggered by IAS cause neuronal activation in the spinal cord. As mentioned earlier, stimulation of primary afferent neurons increases the secretion of CX_3_CL1 into the DRG and spinal cord microenvironment, leading to the activation of microglia that through communication with astrocytes and neurons support an effective neuroimmune communication accounting for neuroinflammation and neuronal activation in the spinal cord. Our data support that this is occurring in DOMS.

In conclusion, this study shows that DOMS caused by IAS depends on spinal cord neuroinflammation. The present data uncover that spinal cord astrocytes and microglia are activated at the peak of DOMS and, through neuron-to-glia as well as glia-to-glia communications, may contribute to increased spinal cord dorsal horn neuronal activity and consequently pain in the post-exercise recovery period. Besides, IAS-induced DOMS depends on NFκB activation, modulation of cytokines (including TNF-α, IL-1β, and IL-10), and oxidative stress in the spinal cord. Thus, this study advances in the understanding of neuroimmune interactions in DOMS pathophysiology at the spinal cord, which may have clinical relevance contributing to the therapy and prophylaxis of DOMS.

## Data Availability

The raw data supporting the conclusions of this article will be made available by the authors, without undue reservation.
