# Supplementary material for: Intense Acute Swimming Induces Delayed-Onset Muscle Soreness Dependent on Spinal Cord Neuroinflammation
Source: Front Pharmacol. 2022 Jan 7;12:734091. doi: 10.3389/fphar.2021.734091 (PMC8776654; doi:10.3389/fphar.2021.734091)
Supplement: Supplementary file 1 [file DataSheet1.PDF]

Intense acute swimming induces delayed onset muscle soreness dependent on spinal cord neuroinflammation

Sergio M. Borghi,[1,2,*] Sylvia K. D. Bussulo,[2] Felipe A. Pinho-Ribeiro,[1] Victor Fattori,[1] Thacyana T. Carvalho,[1] Fernanda S. Rasquel-Oliveira[1], Tiago H. Zaninelli,[1] Camila R. Ferraz,[1] Antônio M. B. Casella,[3] Fernando Q. Cunha,[4] Thiago M. Cunha,[4] Rubia Casagrande,[5] and Waldiceu A. Verri Jr[1,*]

[1]Departamento de Ciências Patológicas, Centro de Ciências Biológicas, Universidade Estadual de Londrina, Rodovia Celso Garcia Cid, Pr 445, Km 380, 86057970 Londrina, Brazil.

[2]Centro de Pesquisa em Ciências da Saúde, Universidade Norte do Paraná, Rua Marselha, 591, Jardim Piza, 86041-140, Londrina, Paraná, Brazil.

[3]Departamento de Ciências da Saúde, Centro de Ciências de Saúde, Universidade Estadual de Londrina, Avenida Robert Koch, 60, 86038-350, Londrina, Paraná, Brazil.

[4]Departamento de Farmacologia, Faculdade de Medicina de Ribeirão Preto, Universidade de São Paulo, Avenida Bandeirantes, 3900, 14049-900, Ribeirão Preto, São Paulo, Brazil.

[5]Departamento de Ciências Farmacêuticas, Centro de Ciências de Saúde, Hospital Universitário, Universidade Estadual de Londrina, Avenida Robert Koch, 60, 86038-350, Londrina, Paraná, Brazil.

[*]Authors to whom correspondence should be addressed: Prof. Sergio M. Borghi, Centro de Pesquisa em Ciências da Saúde, Universidade Norte do Paraná, Rua Marselha, 591, Jardim Piza, 86.041-140, Londrina, Paraná, Brasil; Tel: + 55 43 3371-7990; Mobile: + 55 43 99918-9216; E-mail address: sergio_borghi@yahoo.com.br or sergio.borghi@kroton.com.br; and Prof. Waldiceu A. Verri Jr, Centro

de Ciências Biológicas Departamento de Ciências Patológicas, Rod. Celso Garcia Cid, Pr 445, Km 380, Postal Code 10.011, 86057-970, Londrina, Paraná, Brasil; Tel. + 55 43 3371-4979; E-mail address: waldiceujr@yahoo.com.br or waverri@uel.br.

Running title: Swimming-induced DOMS and spinal neuroinflammation.

SUPPLEMTARY FILES

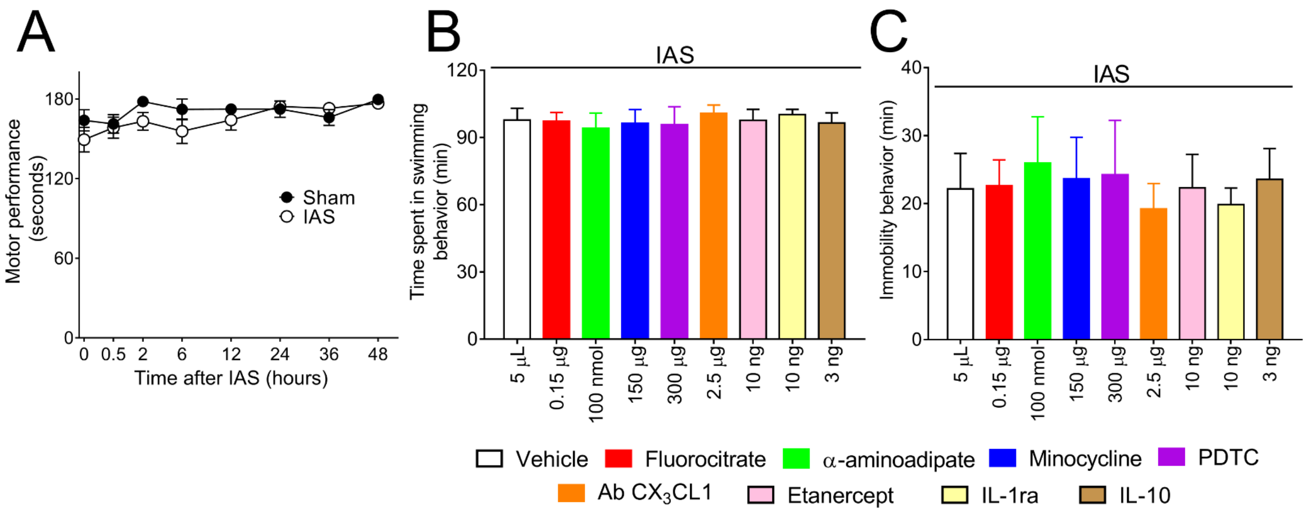

FIGURE S1. Evaluation of motor performance after IAS, and mobility/immobility behavior during IAS session. **(A)** Motor performance in the rota-rod test was evaluated in sham and IAS mice 2-48 h after swimming session. Doses of i.t. treatments performed during the study (vehicle, 5 µL; fluorocitrate, 0.15 µg; α-aminoadipate, 100 nmol; minocycline, µg; PDTC, 300 µg; Ab CX_3CL1, 2.5 µg; etanercept, 10 ng; IL-1ra, 10 ng; rmIL-10, 3 ng) did not significantly affect **(B)** time spent in swimming behavior or **(C)** immobility behavior during the IAS session. Results are presented as motor performance (in seconds), and as time spent in swimming behavior and immobility behavior, both in minutes (*n* = 4 mice per group per experiment and is representative of two independent experiments).

Compound	Application	Manufacturer	Dose
Saline solution (NaCl 0.9%)	Vehicle	Frenesius Kabi Brasil Ltda, Aquiraz, CE, Brazil	5 µL
L-2-aminoadipic acid (α-aminoadipate)	Astrocyte inhibitor	Sigma-Aldrich, St. Louis, MO, USA	10-100 nmol
Minocycline hydrochloride	Microglia inhibitor	Sigma-Aldrich, St. Louis, MO, USA	15-150 µg

DL-flourocitric acid barium salt (fluorocitrate)	Astrocyte inhibitor	Sigma-Aldrich, St. Louis, MO, USA	0.05-0.45 µg
Pyrrolidine dithiocarbamate (PDTC)	NFκB activation inhibitor	Sigma-Aldrich, St. Louis, MO, USA	30-300 µg
Mouse neutralizing antibody anti-CX_3CL1 (polyclonal goat IgG; F472)	Neutralize CX_3CL1	R&D Systems, Minneapolis, MN, USA	0.25-2.5 µg
Isotype-matched antibody (control IgG; AB-108-C)	Control antibody	R&D Systems, Minneapolis, MN, USA	µL/4 µL in saline
Etanercept [Enbrel®, soluble tumor necrosis factor receptor 2 (TNFR2)]	Soluble receptor to inhibit TNFα activity	Wyeth, São Paulo, SP, Brazil	10-100 ng
IL-1 receptor antagonista (IL-1ra)	An antagonist of IL-1R.	National Institute for biological Standards and Control (NIBSC), South Mimms, Hertfordshire, UK	10-100 ng
Recombinant mouse IL-10 (rmIL-10)	For treatment with IL-10	eBioscience, San Diego, CA, USA	1-3 ng

Table S1. Chemicals used in the study.

Antibody	Catalog	Dilution	Manufacturer
GFAP	#180063	1:500	Invitrogen, Life Technologies, 261 Carlsbad, CA, USA
Iba-1	#PA5-27436	1:500	Invitrogen, Life Technologies, Carlsbad, CA, USA
Alexa Fluor 488	#A-110088	1:1000	Thermo Fischer Scientific, Waltham, MA, USA
pNFκB	#sc-136548	1:200	Santa Cruz Biotechnology, Inc., Dallas, TX, USA
IgG-HRP	#sc-2005	1:1000	Santa Cruz Biotechnology, Inc., Dallas, TX, USA
c-Fos	#ab190289	1:500	Abcam Plc., Cambridge, UK
NeuN	#MAB377	1:1000	Merck Millipore, Burlington, MA, USA
Alexa Fluor 488	#A11001	1:1000	Thermo Fischer Scientific, Waltham, MA, USA
Alexa Fluor 647	#A32733	1:1000	Thermo Fischer Scientific, Waltham, MA, USA

Table S2. Immunofluorescence antibodies used in the study.

Target gene	Forward	Reverse
Gfap	5´-GGCGCTCAATGCTGGCTTCA-3´	5´-TCTGCCTCCAGCCTCAGGTT-3´
Iba1	5´-ATGGAGTTTGATCTGAATGGAAAT-3´	5´-TCAGGGCAGCTCGGAGATAGCTTT-3´
Cx3cr1	5´-CACCATTAGTCTGGGCGTCT-3´	5´-GATGCGGAAGTAGCAAAAGC-3´
Tnfα	5´-TCTCATCAGTTCTATGGCCC-3´	5´-GGGAGTAGACAAGGTACAAC-3´
Il1β	5′-GAAATGCCACCTTTTGACAGTG-3′	5′-TGGATGCTCTCATCAGGACAG-3′
Il10	5´-TCTCATCAGTTCTATGGCCC-3´	5´-GGGAGTAGACAAGGTACAAC-3´
cfos	5´-GGGCTGCACTACTTACACGT-3´	5´-TGCCTTGCCTTCTCTGACTG-3´
β-actin	5´-AGCTGCGTTTTACACCCT TT-3´	5´-AAGCCATGCCAATGTTGTCT-3´

Table S3. Mouse mRNA primers used for RT-qPCR.

The ARRIVE Guidelines Checklist

Animal Research: Reporting In Vivo Experiments

Carol Kilkenny[1], William J Browne[2], Innes C Cuthill[3], Michael Emerson[4] and Douglas G Altman[5]

[1]The National Centre for the Replacement, Refinement and Reduction of Animals in Research, London, UK, [2]School of Veterinary Science, University of Bristol, Bristol, UK, [3]School of Biological Sciences, University of Bristol, Bristol, UK, [4]National Heart and Lung Institute, Imperial College London, UK, [5]Centre for Statistics in Medicine, University of Oxford, Oxford, UK.

	ITEM	RECOMMENDATION	Section/ Paragraph
Title	1	Provide as accurate and concise a description of the content of the article as possible.	Title page.
Abstract	2	Provide an accurate summary of the background, research objectives, including details of the species or strain of animal used, key methods, principal findings and conclusions of the study.	Abstract.
INTRODUCTION			
Background	3	a. Include sufficient scientific background (including relevant references to previous work) to understand the motivation and context for the study, and explain the experimental approach and rationale.<br>b. Explain how and why the animal species and model being used can address the scientific objectives and, where appropriate, the study's relevance to human biology.	Paragraphs 1-3 of the introduction.

Objectives	4	Clearly describe the primary and any secondary objectives of the study, or specific hypotheses being tested.	Introduction and materials and methods.
METHODS			
Ethical statement	5	Indicate the nature of the ethical review permissions, relevant licences (e.g. Animal [Scientific Procedures] Act 1986), and national or institutional guidelines for the care and use of animals, that cover the research.	Materials and methods, animal section.
Study design	6	For each experiment, give brief details of the study design including:<br>a. The number of experimental and control groups.<br>b. Any steps taken to minimise the effects of subjective bias when allocating animals to treatment (e.g. randomisation procedure) and when assessing results (e.g. if done, describe who was blinded and when).<br>c. The experimental unit (e.g. a single animal, group or cage of animals).<br>A time-line diagram or flow chart can be useful to illustrate how complex study designs were carried out.	Materials and methods, animals and general experimental procedures sections; and Figure 1.
Experimental procedures	7	For each experiment and each experimental group, including controls, provide precise details of all procedures carried out. For example:<br>a. How (e.g. drug formulation and dose, site and route of administration, anaesthesia and analgesia used [including monitoring], surgical procedure, method of euthanasia). Provide details of any specialist equipment used, including supplier(s).<br>b. When (e.g. time of day).<br>c. Where (e.g. home cage, laboratory, water maze).<br>d. Why (e.g. rationale for choice of specific anaesthetic, route of administration, drug dose used).	Materials and methods, methods for intrathecal (i.t.) injections and general experimental procedure sections, and Table S1.
Experimental animals	8	a. Provide details of the animals used, including species, strain, sex, developmental stage (e.g. mean or median age plus age range) and weight (e.g. mean or median weight plus weight range).<br>b. Provide further relevant information such as the source of animals, international strain nomenclature, genetic modification status (e.g. knock-out or transgenic), genotype, health/immune status, drug or test naïve, previous procedures, etc.	Materials and methods, animals section.
Housing and husbandry	9	Provide details of:<br>a. Housing (type of facility e.g. specific pathogen free [SPF]; type of cage or housing; bedding material; number of cage companions; tank shape and material etc. for fish).<br>b. Husbandry conditions (e.g. breeding programme, light/dark cycle, temperature, quality of water etc for fish, type of food, access to food and water, environmental enrichment).<br>c. Welfare-related assessments and interventions that were carried out prior to, during, or after the experiment.	Materials and methods, animals section.

Sample size	10	a. Specify the total number of animals used in each experiment, and the number of animals in each experimental group.<br>b. Explain how the number of animals was arrived at. Provide details of any sample size calculation used.<br>c. Indicate the number of independent replications of each experiment, if relevant.	Materials and methods, general experimental procedure section, Figure 1 and figure legends.
Allocating animals to experimental groups	11	a. Give full details of how animals were allocated to experimental groups, including randomisation or matching if done.<br>b. Describe the order in which the animals in the different experimental groups were treated and assessed.	Materials and methods, methods for intrathecal (i.t.) injections and general experimental procedure sections.
Experimental outcomes	12	Clearly define the primary and secondary experimental outcomes assessed (e.g. cell death, molecular markers, behavioural changes).	Introduction and materials and methods.
Statistical methods	13	a. Provide details of the statistical methods used for each analysis.<br>b. Specify the unit of analysis for each dataset (e.g. single animal, group of animals, single neuron).<br>c. Describe any methods used to assess whether the data met the assumptions of the statistical approach.	Materials and methods, statistical analysis section.
RESULTS			
Baseline data	14	For each experimental group, report relevant characteristics and health status of animals (e.g. weight, microbiological status, and drug or test naïve) prior to treatment or testing. (This information can often be tabulated).	Materials and methods and results.
Numbers analysed	15	a. Report the number of animals in each group included in each analysis. Report absolute numbers (e.g. 10/20, not 50%[2]).<br>b. If any animals or data were not included in the analysis, explain why.	Materials and methods and figure legends.
Outcomes and estimation	16	Report the results for each analysis carried out, with a measure of precision (e.g. standard error or confidence interval).	Results section and figure legends.
Adverse events	17	a. Give details of all important adverse events in each experimental group.<br>b. Describe any modifications to the experimental protocols made to reduce adverse events.	N/A.
DISCUSSION			

Interpretation/ scientific implications	18	a. Interpret the results, taking into account the study objectives and hypotheses, current theory and other relevant studies in the literature.<br>b. Comment on the study limitations including any potential sources of bias, any limitations of the animal model, and the imprecision associated with the results[2].<br>c. Describe any implications of your experimental methods or findings for the replacement, refinement or reduction (the 3Rs) of the use of animals in research.	Paragraphs 1-8 of the discussion section.
Generalisability/ translation	19	Comment on whether, and how, the findings of this study are likely to translate to other species or systems, including any relevance to human biology.	Last paragraph of the discussion section.
Funding	20	List all funding sources (including grant number) and the role of the funder(s) in the study.	Funding section.

The ARRIVE Guidelines Checklist.
